# New Perspectives on Sex Steroid Hormones Signaling in Cancer-Associated Fibroblasts of Non-Small Cell Lung Cancer

**DOI:** 10.3390/cancers15143620

**Published:** 2023-07-14

**Authors:** Chihiro Inoue, Yasuhiro Miki, Takashi Suzuki

**Affiliations:** Department of Anatomic Pathology, Tohoku University Graduate School of Medicine, Sendai 980-8575, Japan; miki@patholo2.med.tohoku.ac.jp (Y.M.); t-suzuki@patholo2.med.tohoku.ac.jp (T.S.)

**Keywords:** estrogen, steroid hormones, cancer-associated fibroblasts, microenvironment, non-small cell lung carcinoma

## Abstract

**Simple Summary:**

Non-small cell lung cancer (NSCLC) is the most common type of lung cancer. Smoking is a major risk factor for NSCLC; however, the incidence of NSCLC among nonsmokers, especially women, is increasing. Therefore, sex hormones are associated with NSCLC progression and their significance has been demonstrated. Cancer-associated fibroblasts, which reside in cancer stroma, are important components that significantly influence cancer progression by interacting with other cells, including cancer cells. We focused on the roles of cancer-associated fibroblasts in hormonal cell–cell interactions in NSCLC and their prospects.

**Abstract:**

The importance of sex hormones, especially estrogen, in the pathogenesis of non-small-cell lung cancer (NSCLC) has attracted attention due to its high incidence among young adults and nonsmokers, especially those who are female. Cancer-associated fibroblasts (CAFs) reside in the cancer stroma and influence cancer growth, invasion, metastasis, and acquisition of drug resistance through interactions with cancer cells and other microenvironmental components. Hormone-mediated cell–cell interactions are classic cell–cell interactions and well-known phenomena in breast cancer and prostate cancer CAFs. In cancers of other organs, including NSCLC, the effects of CAFs on hormone-receptor expression and hormone production in cancer tissues have been reported; however, there are few such studies. Many more studies have been performed on breast and prostate cancers. Recent advances in technology, particularly single-cell analysis techniques, have led to significant advances in the classification and function of CAFs. However, the importance of sex hormones in cell–cell interactions of CAFs in NSCLC remains unclear. This review summarizes reports on CAFs in NSCLC and sex hormones in cancer and immune cells surrounding CAFs. Furthermore, we discuss the prospects of sex-hormone research involving CAFs in NSCLC.

## 1. Introduction

**Figure 1:**
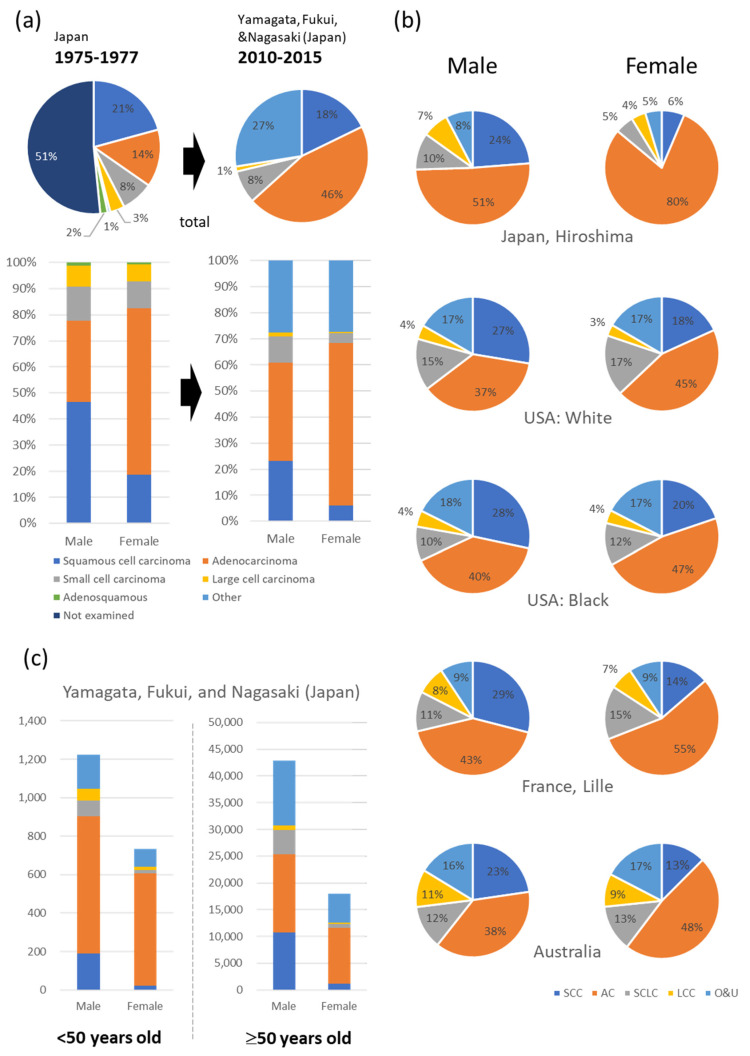
Different histological types of lung cancer based on sex. (**a**) Japanese data, similar to other countries, demonstrate a decline in squamous cell carcinoma and an increase in adenocarcinoma. The decrease in squamous cell carcinoma is particularly significant in males [5,6]. (**b**) Global data indicates a higher incidence of adenocarcinoma in women compared to men [1]. (**c**) The number of lung cancer cases and their histological types in younger (<50 years old) and older adults (≥50 years old) [5]. AC, adenocarcinoma; LCC, large cell carcinoma; NE, not estimable; O & U, other types of carcinoma and carcinoma unspecified; SCC, squamous-cell carcinoma; SCLC, small cell lung carcinoma.

Non-small cell lung cancer (NSCLC) accounts for a large proportion of the increasing number of patients with lung cancer and deaths attributable to lung cancer. The incidence of lung cancer is diverging between men and women, with a decrease in men and an increase in women [1,2,3,4]. In patients in their 70s and 80s, the predominant histological type was squamous cell carcinoma but now it is adenocarcinoma (Figure 1a) [1,2,5,6]. The rate of adenocarcinoma is higher in women than in men (Figure 1b) and is especially higher in younger women than in older women (Figure 1c) [1,5]. The number of adenocarcinomas has plateaued in men; however, both the number and rate of adenocarcinomas continue to increase in women [2,4]. Smoking is the leading cause of lung cancer. While several reports have indicated that women are at a higher risk of lung cancer from smoking [7,8], others have indicated that there is no sex difference in cancer risk among smokers [9]. Estrogen, a female hormone, has been reported to increase the risk of carcinogenesis in female smokers by enhancing the toxic metabolites of benzo(a)pyrene in tobacco smoke through an increase in CYP11A1 and CYP11B1 [10,11]. Although smoking is a risk factor for lung cancer, the incidence of lung cancer unrelated to smoking is increasing worldwide, making it an important health issue [1,3,4]. More patients with lung cancer who had never smoked and were under 50 years old have been found to be women [3,4,12,13]. However, the prognosis after chemotherapy is better in women than in men [14,15]. Additionally, driver mutations such as *epidermal growth factor receptor (EGFR)* mutations, *anaplastic lymphoma kinase (ALK)* translocations, *ROS proto-oncogene 1, and receptor tyrosine kinase (ROS1)* translocations are more common among women [16,17,18]. Additionally, molecularly targeted therapies, such as epidermal growth factor receptor tyrosine kinase inhibitors (EGFR-TKIs) and immune-checkpoint inhibitors, have been used for treatment; however, the acquisition of resistance to these therapies is a serious problem [19,20]. Therefore, therapeutic efficacy and novel therapeutic targets are required. Therefore, it is necessary to improve our understanding of the effects of estrogen and other sex hormones on carcinogenesis, progression, and resistance to lung cancer treatment.

Cancer-associated fibroblasts (CAFs) reside in cancer tissues and have been demonstrated to influence cancer growth and invasion of various organs, including lung cancer, as well as to promote resistance to drug therapies such as EGFR-TKIs; therefore, CAFs have attracted attention as new therapeutic targets. CAFs can interact with their microenvironmental components, such as cancer cells, immune cells, and endothelial cells, via cytokines, chemokines, extracellular matrix, microRNAs (miRNAs), and long noncoding RNA (lncRNAs) in extracellular vesicles. CAFs are heterogeneous cell populations; because of their origin and plasticity, they can change their features by interacting with surrounding cells, by environmental effects such as hypoxia, and by the rigidity of their scaffold. Histologically, CAFs were observed as spindle cells within the fibrous stroma. Currently, no specific marker protein or gene has been identified to exclusively characterize CAFs. Instead, a combination of proteins or genes, such as α-smooth muscle actin (αSMA), fibroblast activating protein (FAP), platelet-derived growth factor receptor α (PDGFRα) and β (PDGFRβ), and podoplanin, is utilized for CAF identification in immunohistochemistry, flow cytometry, and transcriptome analysis [21,22]. Among these markers, αSMA is the most commonly used CAF marker, and a high abundance of αSMA-expressing CAFs in NSCLC tissues has been associated with a poor prognosis [23,24,25]. Recently, genomics and proteomics at the single-cell level and spatial profiling have revealed the characteristics of CAFs, and many researchers have attempted to classify CAFs [22,26,27,28,29]. Additionally, there is an increasing number of reports on the relationship between CAFs and other microenvironmental components, particularly the interaction between immune cells and CAFs, their effects on the efficacy of immune-checkpoint inhibitors, the interaction between the extracellular matrix and CAFs, and cell-to-cell interactions via miRNAs and lncRNAs. Research on organoid cultures, which mimic the close interaction between cancer and other microenvironmental components, is also growing [30]. Hormone-regulated cell–cell interactions are classic and representative cell–cell interactions that have been the focus of CAF–tumor interactions, especially in breast cancer; however, the role of sex hormones in CAFs of NSCLC remains unclear. CAFs, recognized as integral components of cancer, hold the potential for elucidating sex-related differences in the development and characteristics of NSCLC, as they exhibit dynamic adaptability within the microenvironment and engage in multifaceted interactions with cancer cells and other constituents via numerous soluble factors. Notably, sex hormones can exert an influence on CAF features and contribute to cell–cell interactions in NSCLC tissues.

In this review, we discuss the role of sex hormones, particularly estrogen, in the interaction of CAFs with cancer cells and other microenvironmental components of lung-cancer tissues.

## 2. Estrogen: A Female Hormone and Its Receptors

Estrogen is a female hormone, and its most potent form is 17β–estradiol (E2), which is converted from testosterone by aromatase (CYP19) or estrone by 17β–hydroxysteroid dehydrogenase type 1 (17βHSD1). Estrogen receptors (ERs) include the classic ligand-dependent transcriptional regulators (ERα and ERβ) and membrane ERs (G–protein-coupled ER [GPER]/G–protein-coupled receptor 30 [GPR30]), which are newer types of ERs that activate signal transduction pathways. ERα and ERβ, when activated by estrogen, undergo translocation from the cytoplasm to the nucleus, where they bind to response elements in DNA. The ER proteins regulate mRNA transcription by recruiting corepressors and coactivators [31,32]. They act on ER response elements (EREs) of target genes, forming homo (αα or ββ) or heterodimers (αβ) [32]. While the affinity of E2 for each receptor is similar, the transactivation functional domain of ERβ is truncated compared to ERα, resulting in lower activity in inducing transcriptional activation [33].

Estrogen primarily induces cell proliferation in NSCLC through nongenomic pathways, activating cAMP, MAPK, and AKT signaling pathways [34,35]. Growth factors, such as epidermal growth factor (EGF), insulin-like growth factor (IGF), and transforming growth factor–α (TGF-α) can activate estrogen receptors even in the absence of estrogen, primarily through the AF-1 domain [32]. GPER, a seven-transmembrane receptor, when activated, promotes cellular proliferation, inversion, growth, and survival via the cAMP/PKA/CREB, RAS/RAF/MEK/ERK, and ERs/ERK/MAPK pathways in lung cancer [36]. Expressions of ERα, ERβ, and GPER have been observed in normal lungs and the importance of ERβ in lung tissues has been emphasized [33,37]. Antiestrogen therapies include medicines that function through different pathways, such as luteinizing hormone-releasing hormone agonist therapy, which acts on the pituitary gland; aromatase inhibitors, which inhibit estrogen synthesis; and ER antagonists, including selective ER downregulators and modulators [38].

## 3. Estrogen Effect on NSCLC Cells: Role of EGFR Mutations and EGFR-TKI

The effects of estrogen on NSCLC have attracted attention because many young patients with NSCLC are female (Figure 1c) (Figure S1) [5,13,39]. A PubMed search using the terms “non-small lung cell tumor” and “estrogen” revealed that in 2000, there were four articles published per year. However, from 2009 to 2020, the number of articles per year increased to an average of 27.6. Expressions of ERs, including ERα, ERβ, and GPER, as well as aromatase, which is an estrogen synthase, have been reported in NSCLC tissue, including men and postmenopausal women, and have been associated with the NSCLC prognosis [40,41,42,43]. Studies involving animal models have also demonstrated the tumor-promoting effects of estrogen [44,45]. ER expression was positively correlated with *EGFR* mutations in lung adenocarcinoma [46,47,48]. The effects of ERs on the efficacy of EGFR-TKIs and resistance acquisition are important because of the high prevalence of EGFR mutations in female patients [16].

Kohno et al. reported that high aromatase mRNA expression is associated with poor prognosis in women, nonsmokers, and patients with *EGFR* mutations [49]. NSCLC cells with *EGFR* mutations have been observed with the coexpression of cytoplasmic and nuclear ERβ and associated with EGFR-TKI resistance. Furthermore, ERβ expression, particularly ERβ1 expression, is associated with therapeutic resistance to EGFR-TKIs [50,51,52,53]. It has been demonstrated that membrane ERs activate EGFR and that the EGFR pathway activates nuclear ERα [54]; however, in contrast to these coactivating relationships between ERα and EGFR, it has been shown that ERβ expression decreases in response to epidermal growth factor (EGF) and increases in response to EGFR-TKIs [55]. Therefore, combination therapy with EGFR-TKIs and ER inhibitors is considered more effective than EGFR-TKIs alone and the combination of EGFR-TKIs and fulvestrant, an ER antagonist, suppresses the proliferation of NSCLC cells with or without *EGFR* mutations [41,55].

The EGFR pathway is not the only pathway that interacts with ERs. ERβ has been reported to interact with testicular orphan nuclear receptors (TR2) and the EGFR pathway in NSCLC [56,57]. ERβ promotes NSCLC invasion and migration by upregulating TLR4 expression and activating the downstream myeloid differentiation factor 88 (myd88)–nuclear factor-κB (NF-κB)–matrix metalloproteinase-2 (MMP-2) signaling axis [58]. Interactions between ER and other pathways may influence the efficacy of molecularly targeted drugs and radiotherapy and estrogen inhibitors, in combination with other therapies, may enhance the antitumor effects. It has also been reported that the inactivation of p16 and ER genes by carcinogenic particulate matter, particularly beryllium metal, results in tumorigenesis in the lungs [59] and that the influence of estrogen on tumorigenesis and tumor progression may be associated with dust exposure and the resulting inflammation.

In contrast to *EGFR* mutations, *TP53* mutations are negatively associated with ER expression [47]. In those with nonfunctional *TP53*, genistein and E2 increased radiosensitivity [60]. The significance of estrogen may vary depending on the type of therapy and genetic mutations harbored by the cancer cells.

Recently, a therapeutic trial involving an aromatase inhibitor for NSCLC in postmenopausal women showed beneficial effects [61]. In this trial, the objective response rate to the aromatase inhibitor was positively correlated with the immunohistochemical expression of aromatase in cancer cells, while no significant correlation was observed with ERa or PgR [61]. Moreover, the intratumoral concentration of estradiol was found to be positively correlated with aromatase expression in ER-positive cases [62]. In an in vitro study, cell proliferation by testosterone was inhibited by the aromatase inhibitor [62]. The impact of hormone replacement therapy, consisting of estrogen and progestin, on the development and prognosis of lung cancer remains controversial, as previous studies have yielded inconsistent results [35]. Hence, estrogen synthesis and metabolism within NSCLC tissues may hold greater significance for tumor progression in postmenopausal women than serum estrogen levels.

Estrogen influences the development and progression of NSCLC and the efficacy of its therapy; furthermore, it is a potential therapeutic target in anticancer therapy. However, genetic mutations in cancer cells, interstitial lung diseases combined with lung cancer, and interactions between ERs and other pathways must be considered when estimating the anticancer effects of estrogen inhibitors.

## 4. Role of Sex Hormones in the Stroma, including CAFs in NSCLC

Banka et al. demonstrated that E2 has the potential to alter the tumor microenvironment, including CAFs and immune cells, promoting tumor progression [63]. Furthermore, when E2-nonresponsive lung cancer cells were implanted in ovariectomized mice, E2 treatment increased metastasis but did not enhance vascularization or tumor growth. Although the effects of sex hormones on the immune system have been reported, there are few reports on the effects of sex hormones on the characteristics of CAFs and the interactions between CAFs and other cells.

**Figure 2:**
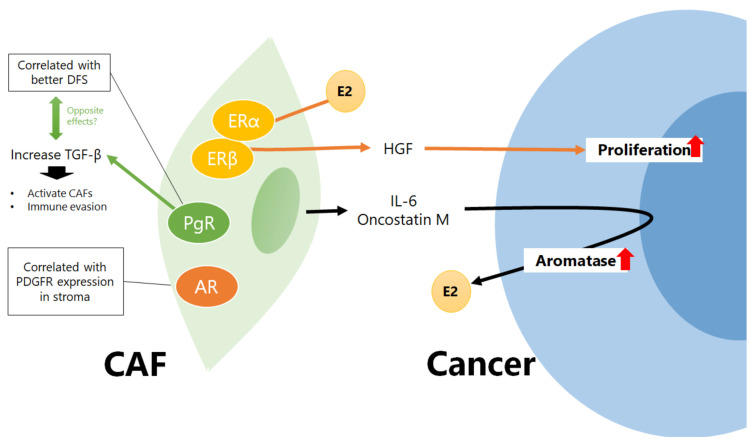
Summary of previously reported roles of sex hormones and their receptors in cancer-associated fibroblasts (CAFs) of nonsmall-cell lung cancer (NSCLC). CAFs in NSCLC have been reported to express estrogen receptor (ER) α and ERβ, progesterone receptor (PgR), and androgen receptor (AR). PgR expression in CAFs was associated with a better disease-free survival rate for patients, despite PgR being reported to enhance CAF activation through increasing transforming growth factor-β (TGF-β). AR expression in CAFs was correlated with the expression of the CAF marker platelet-derived growth factor receptor (PDGFR). Estradiol (E2) enhanced hepatocyte growth factor (HGF) secretion of CAFs and promoted cancer-cell proliferation. Soluble factors secreted from CAFs increased aromatase and E2 production in cancer cells. IL, interleukin.

In 2002, Stabile et al. first reported that ERα and ERβ are expressed in fibroblasts in NSCLC tissue and that both cancer cells and fibroblasts showed enhanced proliferation when treated with E2. Furthermore, the treatment of lung fibroblasts with E2 increased the secretion of hepatocyte growth factor (HGF) from CAFs (Figure 2) [64]. HGF secretion by CAFs is one of the mechanisms underlying CAF-induced EGFR-TKI resistance in NSCLC [65]. Estrogen may induce EGFR-TKI resistance in NSCLC cells by acting not only on cancer cells but also on CAFs. In 2010, Miki et al. focused on the role of the stroma in estrogen synthesis in NSCLC tissues. Intratumoral stromal cells derived from lung cancer tissues did not express aromatase; however, they produced oncostatin M and interleukin (IL)-6, which induced aromatase gene expression in lung adenocarcinoma cell lines (Figure 2) [66], indicating the importance of intratumoral estrogen production induced by CAFs in NSCLC tissues as well as estrogen produced by the ovaries. It is of great interest to understand how CAFs affect sex-hormone synthesis and metabolic pathways through interactions with cancer cells and other microenvironmental components; therefore, comprehensive analyses are required.

Next, focusing on the effects of estrogen on fibroblasts in the lungs, it has been reported that E2 enhances bleomycin-induced inflammatory cytokine production and collagen deposition in rat lungs [67]. Elliot et al. reported that ERα expression was increased in usual interstitial pneumonia (UIP)/idiopathic pulmonary fibrosis (IPF) and interstitial pneumonia tissues of bleomycin-treated mice and that pulmonary fibrosis was reduced by ERα inhibitors in bleomycin-treated mice; however, ERα expression was not significantly different between normal and interstitial pneumonia tissues [68]. A positive feedback mechanism, in which estrogen produced by cancer cells promotes ERα-dependent fibrosis and increases cytokine production by fibroblasts, could exist, thus further promoting estrogen production by cancer cells. However, it has been reported that estrogen suppresses fibrosis in the lungs [69]; therefore, further investigations using three-dimensional cultures or animal models are necessary to consider estrogen concentrations in tissues and cell–cell interactions.

Skjefstad et al. performed a study on sex hormones other than ERs and reported that progesterone receptor (PgR) expression in the lung-cancer stroma was correlated with better disease-free survival regardless of sex; however, stromal PgR expression was lower in cancer tissues than in normal tissues [70]. In contrast, in women, PgR expression in cancer cells was associated with poor prognosis, indicating that the significance of PgR expression in cancer cells and stroma is different. In addition, there was a correlation between ERβ and AR expression in the stroma. The expressions of ERβ and AR in the stroma were correlated with the expressions of their respective receptors in cancer cells. Furthermore, AR expression in the stroma is correlated with PDGFR expression [71]. A previous study reported that PgR was positive, but that AR and ERα were negative, in UIP tissues [72]; however, this result was not consistent with the results of the study by Elliot et al. [68]. Progesterone promotes fibrosis in BLM-induced lung injury in mice by enhancing the expression of α–SMA, a marker of activated fibroblasts, and TGF–β, which activates fibroblasts [73]. Since PgR expression in the cancer stroma is a good prognostic marker [70], PgR-positive CAFs may be a type of tumor-suppressive CAF and not quiescent fibroblasts.

Androgens delay surfactant production and lung maturation during development by affecting pulmonary fibroblasts. Pulmonary fibroblasts express 17βHSD type 2 (17βHSD2) and 5α–reductase with a marked substrate preference for androstenedione, which is the product of 17βHSD2. Furthermore, 17βHSD2 and 5α–reductase decreased androgen levels, including testosterone and 5α–dihydrotestosterone, which are the most potent androgens [74]. Since CAFs induce the expression of aromatase, which converts androgens to estrogens in lung cancer cells [66], androgen levels in lung cancer tissues may be maintained at low levels, and testosterone may have a weaker effect on CAFs than on estrogen. The significance of AR expression in CAFs in NSCLC should be examined in conjunction with androgen concentration in lung cancer tissues. Thus, a detailed study on the significance of AR and PgR expression in CAFs and ERs, along with the concentration of each sex hormone and the expression of synthetic enzymes, is warranted.

## 5. Influence of Female Hormones on Angiogenesis in NSCLC

Sex hormones directly affect the cancer microenvironment and indirectly regulate the secretion of soluble factors by cancer cells. Estrogen is known to affect tumor progression via angiogenesis. Treatment of male mice with E2 increases the expression of vascular endothelial growth factor (VEGF), phospho-NF-κB, and IL-17A [57]. E2 promotes tumor development and increases lymphangiogenesis, angiogenesis, VEGF-A, and basic fibroblast growth factor (bFGF) levels in the lung tumors of female mice through an ERα-dependent pathway [75]. Progesterone also influences angiogenesis. PgR expression in lung cancer is lower than in benign tissues; however, the combination of estrogen and progestin increases tumor VEGF secretion in vitro. Additionally, conditioned media from progestin-treated NSCLC cells promote human vascular endothelial cell proliferation [76]. Furthermore, it has been reported that membrane PRα, which is highly expressed in lung adenocarcinoma cells, promoted the activation of cAMP/JAK/STAT3 signaling, increased hypoxia-inducible factor-1α (HIF1α)–induced VEGF secretion, enhanced angiogenesis, and promoted human vascular endothelial cell migration and tube formation under hypoxia [77]. Thus, estrogen and progesterone may promote the growth and metastasis of NSCLC cells by enhancing angiogenesis.

Bevacizumab, an anti-vascular endothelial growth factor (VEGF) antibody, combined with platinum-based chemotherapy, is a commonly employed treatment for advanced NSCLC. Patel et al. demonstrated in an NSCLC mouse model that estrogen increased VEGF and PDGF-BB levels, promoted pericyte coverage and myeloid recruitment, and, consequently, led to resistance against VEGF-targeted therapies [78]. However, contradicting these findings, Wakelee et al. reported that women under 60 years old, including premenopausal women, experienced greater survival benefits from bevacizumab compared to postmenopausal women over 60 years old [14]. It is essential to consider nonestrogenic effects as well to gain a comprehensive understanding of the mechanisms underlying bevacizumab resistance in patients with NSCLC.

## 6. Influence of Female Hormones on Tumor Immunity in NSCLC

The immune system is influenced by sex hormones, such as estrogen, progesterone, and androgens [79]. Immune-checkpoint inhibitors are widely used for lung cancer treatment [1]; therefore, the number of studies on the effects of estrogen on tumor immunity in lung cancer is increasing and data analyses using public databases have shown that ER and/or PgR expression in cancer tissues correlate with immune cell infiltration. Zhu et al. reported that ERα expression is positively correlated with the infiltration of dendritic cells, macrophages, neutrophils, B cells, CD4+ T cells, and CD8+ T cells; however, there is no correlation between ERα expression and the prognosis [80]. Oh et al. analyzed RNA seauencing data from The Cancer Genome Atlas and demonstrated that increased ERα was associated with high expression of immune-checkpoint markers, whereas increased PgR was associated with high levels of TGF–β genes [81]. TGF–β, which is associated with PgR, is a well-known fibroblast activator that may stimulate CAF activation.

Estrogen enhances tumor immune evasion by remodeling tumor immunity into a protumorigenic state. Among patients with pT1a lung adenocarcinomas, those with ERα expression had higher recurrence rates. ERα expression was also positively correlated with the number of Foxp3-positive lymphocytes and IL-7 receptor [82]. Nonsteroidal anti-inflammatory drugs, such as aspirin, have been reported to decrease circulating E2 levels in postmenopausal women [83,84]. In a mouse model of tobacco carcinogenesis, a combination of nonsteroidal anti-inflammatory drugs and aromatase inhibitors resulted in smaller tumors and decreased circulating E2 and IL-6 levels, tumor-infiltrating macrophages, and the expression of tumor Ki-67, phospho–MAPK, phospho–STAT3, and IL-17A [57]. Estrogen inhibitors restored macrophage phagocytic activity in a mouse model of lung cancer with *KRAS G12C* and *TP53* mutations, similar to tobacco-induced lung adenocarcinoma in humans [85]. Chen et al. reported that E2 increased DNA methyltransferase 1 (DNMT1) expression and enhanced the methylation of the *TP53* promoter in lung-cancer cells of female mice harboring the *EGFR L858R* mutation. *TP53* knockout in mice with *EGFR L858R*-induced lung cancer increases M2 macrophage polarization by increasing C–C motif chemokine ligand 5 (CXCL5) expression and decreasing growth differentiation factor 15 (GDF15) expression. These results suggest that E2 may cause the tumor microenvironment to be enriched in M2 macrophages through p53 suppression by DNMT1 [86].

Estrogen has also been reported to contribute to tumor-suppressing immunity. In a female-mouse lung-cancer model with a *K-ras* mutation and conditional deletion of *Stat3*, ER blockade increased the levels of immune suppression markers such as IL-6, CXCL2, and Foxp3 compared with those in control groups, and decreased the expression of immune genes, such as *Ifng*, *Tbx21*, and *Gzmb*, related to Th1 differentiation and the cytotoxic antitumor response. This indicates that estrogen may induce an antitumor immune response [87]. The differential effects of estrogens on immune function reflect not only the estrogen concentration but also the immune cell density, distribution, and type of ERs in immune cells [79]. Tumor features should be considered in tumor immunity. For example, in a mouse model of colon cancer, estrogen was reported to decrease PD-L1 expression and CAFs in cancer cells but it increased the percentage of M1 macrophages in tumor-associated macrophages [88]. However, it has been noted that colon-cancer MC38 cells used in the aforementioned experiment can be used as a model for hypermutated/microsatellite-unstable tumors [89]. These results may have been affected by neoantigen-mediated immune activation.

In addition to hormones, the type of genetic mutation of cancer cells, mutation burden of cancer cells, and smoking status may also influence the production of cytokines and chemokines in cancer cells and influence microenvironmental components, such as immune cells and CAFs. Future studies using human NSCLC specimens are warranted to compare various factors, such as the amount, density, and type of tumor-infiltrating immune cells, clinical factors (including smoking history), genetic mutations of cancer cells, estrogen levels, and ER expression, to elucidate the influence of estrogen on tumor immunity in NSCLC.

## 7. Sex Hormones and miRNAs and lncRNAs in NSCLC

CAFs release extracellular vesicles containing miRNAs and influence cancer progression [90]. For example, exosomal miR-200 secreted by CAFs in lung cancer inhibits the migration, invasion, and epithelial–mesenchymal transition of NSCLC cells [91]. In breast cancer, estrogen administration to CAFs alters miRNA expression patterns and CAFs in skin metastases alter miRNA expression threefold more than in primary breast cancer [92]. However, there are few reports on the relationships between sex hormones, miRNAs, and lncRNAs in NSCLC. Enhancer RNAs (eRNA), a group of lncRNAs derived from enhancer regions, have been reported to be downstream of sex-hormone receptors such as ER and AR. Analysis of the RNA dataset showed that one eRNA, TBX5-AS1, was positively correlated with AR expression; male squamous-cell carcinoma patients with high TBX5-AS1 expression had poor prognoses and the group with high TBX5-AS1 expression had a higher percentage of resting memory CD4+ T cells and fewer follicular helper T cells [93]. Further in vitro and in vivo studies are required to elucidate the mechanism through which this eRNA is associated with AR expression. miR-224-5p promotes NSCLC invasion and metastasis and suppresses AR [94]. It has been reported that miR-224 produced by CAF induced upregulation of the SIRT3–AMPK–mTOR–HIF-1α axis in cancer cells and promoted proliferation and invasion in NSCLC [95]. The miRNAs and lncRNAs produced by CAF may alter the sensitivity of NSCLC to sex hormones and CAFs in breast cancer [92]. Conversely, sex hormones may influence the production of miRNAs and lncRNAs in CAFs and cancer cells.

## 8. Prospects for Sex-Hormone Research of CAFs of NSCLC

As previously mentioned, there are few reports on the effects of sex hormones on the interactions between CAFs and lung cancer cells. There are various reports on how the interactions between CAFs and cancer cells affect the expression of sex hormones and their receptors, not only in hormone-sensitive cancers that express sex-hormone receptors in cancer cells and are treated with antihormonal therapy (such as breast, endometrial, ovarian, and prostate cancers) but also in cancers that are not widely known to express sex-hormone receptors (such as stomach and colon cancers). However, reports on the effects of sex hormones on CAFs in other organs may not be directly applicable to CAFs in lung cancer because the characteristics of fibroblasts and cancer cells differ depending on the organ and disease [21,96]. Therefore, it is valuable to examine whether what has been proven for fibroblasts in other organs can also be proven for lung-cancer CAFs. Drawing from existing research on the roles of sex hormones in CAFs in various tumor types, we highlight several areas that merit further investigation regarding CAFs in NSCLC.

### 8.1. Influence of CAFs in Hormonal Therapy Resistance in NSCLC

Antihormonal therapy for NSCLC may become more common in the future because of the safety and benefits that the aromatase inhibitor combination therapy for NSCLC has demonstrated [61]. The association between hormonal therapy resistance and CAFs in NSCLC should be considered because CAFs are known to affect the efficacy of hormone therapy in breast and prostate cancers by modulating estrogen and androgen sensitivity of cancer cells, maintaining a hormone-dependent microenvironment, or affecting the expression and activation of hormone receptors [97,98,99,100,101]. For example, in prostate cancer, coculturing cancer cells with fibroblasts has been shown to maintain prostate-specific antigen production, stimulated by factors such as EGF, IGF1, and IL-6 released by CAFs following androgen deprivation therapy [97]. Similarly, in breast cancer, coculturing cancer cells with fibroblasts led to enhanced ER transactivation but reduced ER expression and apoptosis rates [99]. CAFs in NSCLC may also influence the efficacy of antihormonal therapies. Interferon-γ (IFN-γ) secreted by CD8-positive T cells has been reported to abolish chemoresistance caused by apoptosis reduction induced by CAFs [102]. When studying resistance to therapies, it is crucial to consider the interactions between each component of the cancer microenvironment, including cancer cells, CAFs, and immune cells.

### 8.2. Effects of AntiSex-Hormone Therapy on the Lung Microenvironment

Hormonal therapies for other diseases may also affect the lung microenvironment. Patients with breast cancer treated with antiestrogen therapy have lower lung cancer incidence and mortality rates [103,104]. In another study involving patients with triple-negative breast cancer (TNBC), which lacks expression of ER, PgR, and HER2, a higher incidence of lung cancer and lower overall survival rates were observed compared to patients with ER and PgR positivity [105]. While antiestrogen therapy is not typically used in patients with TNBC, lung cancer in patients with TNBC might not benefit from such therapy. The potential inhibitory effects of antiestrogen therapy on estrogen-dependent carcinogenesis in epithelial cells and stroma need further investigation, as the underlying mechanisms are still unclear. Two important considerations for understanding how estrogen inhibitors suppress lung cancer development are as follows: first, selective estrogen receptor modulators such as tamoxifen, commonly used in breast cancer treatment, can act as agonists or antagonists of estrogen receptors depending on the cell type and promoter elements [32,106,107]. Therefore, their effects on normal lung tissues should be explored. Secondly, tamoxifen has been associated with an increased risk of lung injury [108,109,110] and does not offer protection against lung injury.

Androgen deprivation therapy (ADT) for prostate cancer reduces the risks of NSCLC and death [111,112]. In these reports, several mechanisms have been proposed, including the induction of cyclin D1 expression in alveolar cells by AR, crosstalk between AR and EGFR, direct effects on lung cancer growth, polarization of macrophage M2, and increased susceptibility to cytotoxic T cells [111,112]. However, the effects of ADT on CAFs have not been considered. After ADT treatment, CAFs secrete neuregulin 1, which promotes resistance in tumor cells through the activation of HER3 [101]. ADT may also affect the microenvironment of other organs, including the lungs.

Anti-sex-hormone therapy administered in other cancers may affect the microenvironment, including fibroblasts and immune cells, which are known to affect tumorigenesis and NSCLC progression. The effects on the microenvironment may differ depending on the original sex-hormone levels and the mechanism of anti-sex hormone therapy.

### 8.3. Sex Hormones and Production of Soluble Factors in Cancer Cells

Sex hormones affect the production of soluble factors by cancer cells, some of which are associated with CAFs. For example, IL-6 and VEGF have been reported to be increased by estrogenic effects in NSCLC mouse models and are known to be produced by CAFs in lung cancer [113]. Estrogen enhances IL-6 production by CAFs in gastric cancer [114]. Estrogen and other sex hormones may influence the production of cytokines and chemokines in NSCLC CAFs because ER is expressed in NSCLC CAFs. Recently, single-cell analysis has been used to classify CAFs, and the mRNA expression of some cytokines and chemokines has been used [115]; however, the relationship between these factors and sex hormones has not been clarified. Therefore, further studies are warranted in this regard.

### 8.4. Role of Sex Hormones in CAF-Mediated Immune Evasion and the Efficacy of Checkpoint Inhibitors

Recently, CAFs have received increasing attention because of their effect on the therapeutic efficacy of immune-checkpoint inhibitors by promoting or suppressing tumor immunity [116]. A recent study has also reported antigen presentation by CAFs [117]. In prostate cancer, AR-negative CAFs are known to surround cancer-cell nests and AR-negative CAFs have been reported to affect cancer growth, invasion, and stemness [118,119]. VEGF-NRP2 signaling has been reported to maintain PD-L1 expression in prostate cancer [120] while VEGF expression is promoted by estrogen, as previously described in Chapter 4. Ellem et al. reported that ERα and ERβ, rather than AR, were predominant in prostate cancer CAFs and that estrogenic effects increased CXCL12 [121]. In contrast, estrogen may affect CXCL12 production by CAFs, therefore, immune evasion because CXCL12 production by FAP-positive CAFs in pancreatic cancer can induce immune evasion [122]. In addition, IFN-γ is a crucial factor to promote PD-L1 expression in cancer cells [123]. In prostate cancer, CAFs have been reported to produce IFN-γ and macrophage colony-stimulating factors in response to the activation of AR pathways [119]; thus, CAFs may promote PD-L1 expression in cancer cells through IFN-γ production. The effects of sex hormones on CAF-mediated tumor immunity and the efficacy of checkpoint inhibitors require further evaluation.

### 8.5. Influence of Tissue Remodeling on Hormone Receptor Expression

CAFs produce various extracellular matrix components, including collagen fibrils and remodel tissues. Sex hormones can induce tissue remodeling in CAFs. In gastric cancer, E2 treatment induces CD147 and IL-6 expression in ER-positive CAFs, increases MMP2 and MMP9 expression, and promotes cancer cell migration and invasion [114,124]. In a mouse breast-cancer model, E2 administration increased the levels of tenascin C, fibronectin 1, periostin, cross-linking enzymes, and collagen [125]. The stiffness of the microenvironment also affects the behavior of cancer cells [126,127]. In breast cancer, soft microenvironments decreased ERα expression [128]. Substrate stiffness has been reported to affect the behavior of cancer cells [129,130,131], with fibroblasts being the main source of extracellular matrices. Therefore, CAFs may also modulate hormone-receptor expression in cancer cells by changing the stiffness of the microenvironment, thereby producing extracellular matrices such as collagen.

## 9. Conclusions and Future Directions

**Figure 3:**
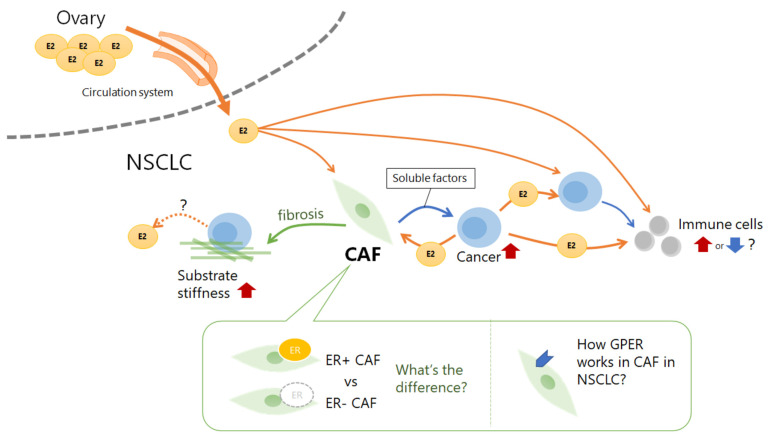
Estrogen production and action in the cancer microenvironment. Cancer-associated fibroblasts (CAFs) in non-small cell lung cancer (NSCLC) tissue are the focus. Estradiol (E2) is produced both inside and outside of NSCLC tissue. The ovary produces estrogen, which is carried to NSCLC tissue by the bloodstream, whereas cancer cells are known to produce estrogen. Estrogen stimulates cancer cells, immune cells, and CAFs. CAFs enhance E2 production in cancer cells by secreting cytokines. Fibrosis by CAFs may increase estrogen production by cancer cells by increasing the substrate stiffness. ER, estrogen receptor; GPER, G-protein-coupled estrogen receptor.

Although there are few reports on the relationship between CAFs and sex hormones in NSCLC tissues, research on this topic, with a focus on interactions, with not only tumor cells but also other microenvironmental components and their effects on therapeutic efficacy, is expected to increase in the future. As summarized in Figure 3, CAFs may play an important role in cell–cell communication through estrogen; however, the influence of estrogen on CAFs in NSCLC remains unclear. Research on sex hormones in the tumor microenvironment has revealed not only the systemic level of hormone synthesis, such as sex hormones regulated by gonads and upstream hormones but also the focal level of synthesis, such as hormones produced in tumor cells or other microenvironment components. These two synthetic methods have complex relationships; therefore, they must be considered separately. Furthermore, in addition to hormone production levels, other important factors that affect hormone synthesis, such as the cell type that produces the hormones, enzymes, cytokines, and chemokines, should also be considered. Therefore, future studies on the role of sex hormones in the CAFs of NSCLC should consider various factors, such as the effects of interactions between receptors and their downstream pathways, background lung conditions, smoking habits, occupational inhalation history, mutational status of NSCLC cells, extracellular matrices, and cytokines produced by CAFs, immune cells, and tumor cells. Comprehensive analyses of cell–cell relationships using three-dimensional reconstruction imaging, mass spectrometry imaging, spatial transcriptomics, and multiple fluorescent immunostaining are effective for understanding the roles of sex hormones in CAFs in NSCLC tissues.

## Supplementary Materials

The following supporting information can be downloaded at: https://www.mdpi.com/article/10.3390/cancers15143620/s1, Figure S1: Age-standadaized rate per 100,000 of the incidence of lung among men and women aged 20−40 (left) and 50 and older (right).
