# Supplementary material for: New Perspectives on Sex Steroid Hormones Signaling in Cancer-Associated Fibroblasts of Non-Small Cell Lung Cancer
Source: Cancers (Basel). 2023 Jul 14;15(14):3620. doi: 10.3390/cancers15143620 (PMC10377312; doi:10.3390/cancers15143620)
Supplement: Supplementary file 1 [file cancers-15-03620-s001.zip › cancers-2401522-supplementary.pdf]

Supplemental figure S1

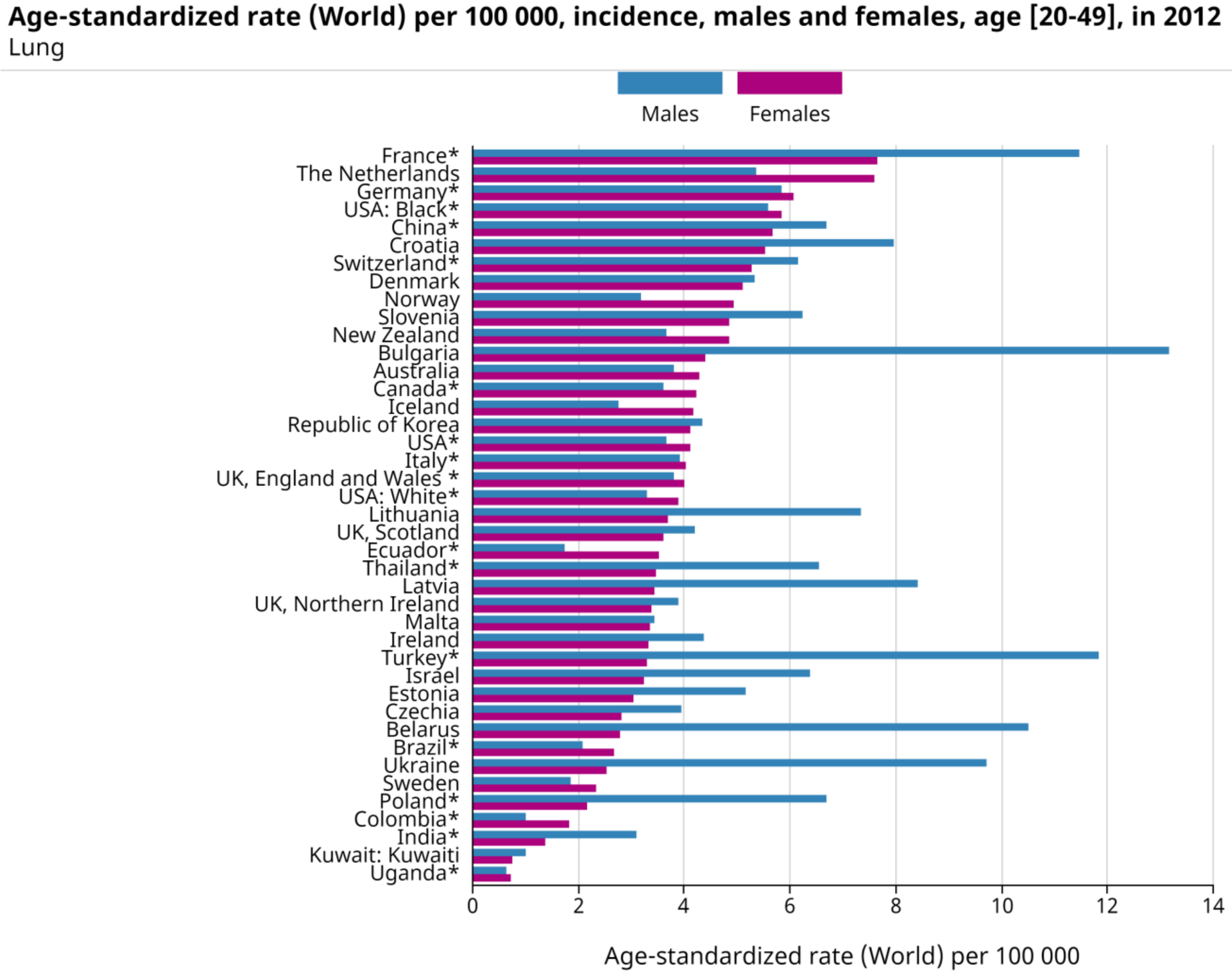

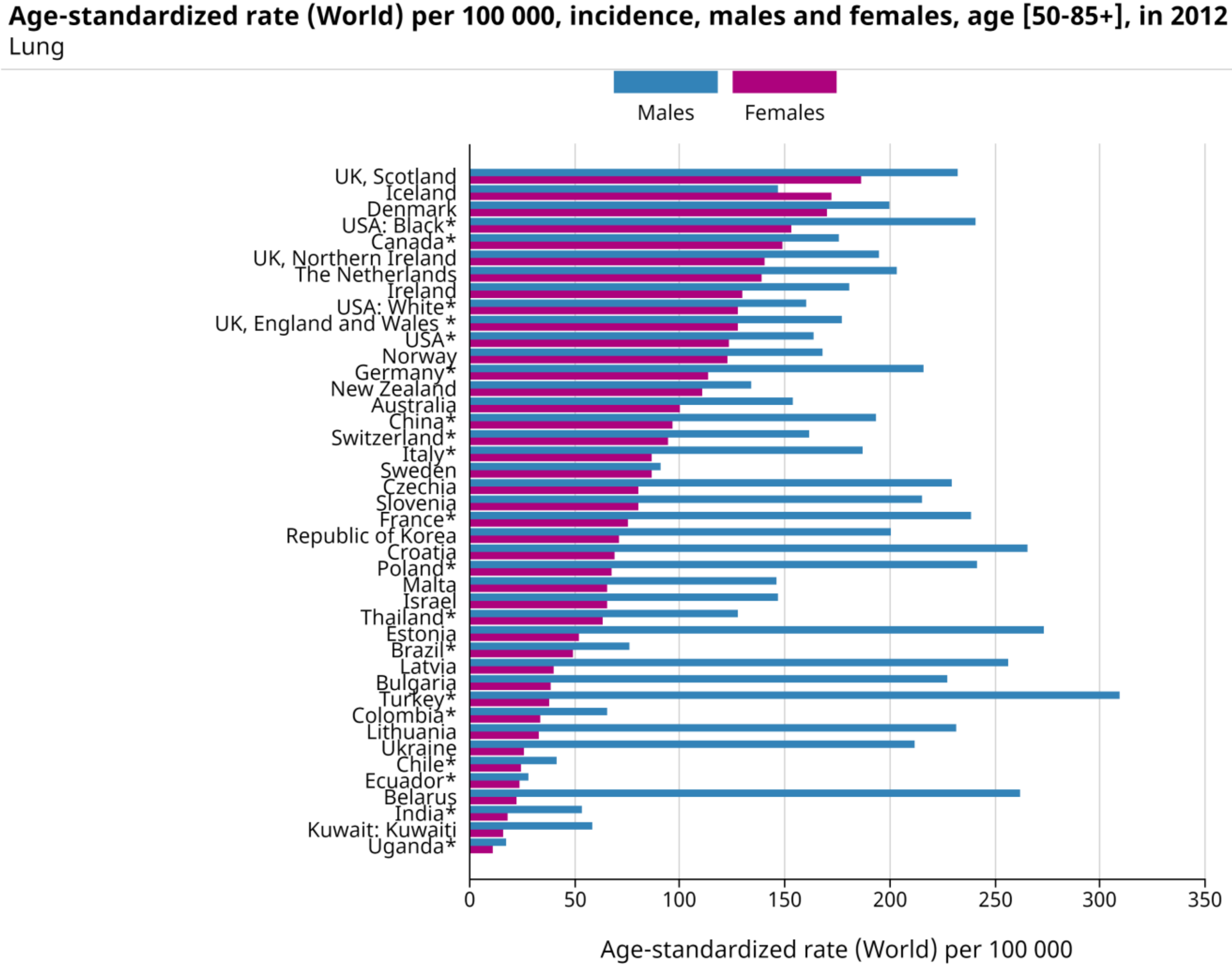

** Subnational data*

Cancer Over time | IARC - All Rights Reserved 2023 - Data version: 1.0

Supplemental figure S1: Age-standadaized rate per 100 000 of the incidence of lung among men and women aged 20-40 (left) and 50 and older (right).

Data source and Graph production: Ervik M, Lam F, Laversanne M, Ferlay J, Bray F (2021). Global Cancer Observatory: Cancer Over Time. Lyon, France: International Agency for Research on Cancer. Available from: https://gco.iarc.fr/overtime , accessed 08 June 2023.
